# Comprehensive analysis of m6A modification lncRNAs in high glucose and TNF-α induced human umbilical vein endothelial cells

**DOI:** 10.1097/MD.0000000000033133

**Published:** 2023-03-10

**Authors:** Li Shan, Mingfei Guo, Yaji Dai, Liangbing Wei, Wei Zhang, Jiarong Gao

**Affiliations:** a Department of Pharmacy, The First Affiliated Hospital of Anhui University of Chinese Medicine, Hefei, China; b Department of Scientific Research, The First Affiliated Hospital of Anhui Medical University, Hefei, China; c Anhui Public Health Clinical Center, Hefei, China; d Department of Pharmacy, Anhui No.2 Provincial People’s Hospital, Hefei, China.

**Keywords:** diabetes, high glucose, HUVECs, lncRNA, N6-methyladenosine, TNF-α

## Abstract

N6-methyladenosine (m6A) RNA methylation, as a reversible epigenetic modification of mammalian mRNA, holds a critical role in multiple biological processes. m6A modification in Long non-coding RNAs (lncRNAs) has increasingly attracted more attention in recent years, especially in diabetics, with or without metabolic syndrome. We investigated via m6A-sequencing and RNA-sequencing the differentially expressed m6A modification lncRNAs by high glucose and TNF-α induced endothelial cell dysfunction in human umbilical vein endothelial cells. Additionally, gene ontology and kyoto encyclopedia of genes and genomes analyses were performed to analyze the biological functions and pathways for the target of mRNAs. Lastly, a competing endogenous RNA network was established to further reveal a regulatory relationship between lncRNAs, miRNAs and mRNAs. A total of 754 differentially m6A-methylated lncRNAs were identified, including 168 up-regulated lncRNAs and 266 down-regulated lncRNAs. Then, 119 significantly different lncRNAs were screened out, of which 60 hypermethylated lncRNAs and 59 hypomethylated lncRNAs. Moreover, 122 differentially expressed lncRNAs were filtered, containing 14 up-regulated mRNAs and 18 down-regulated lncRNAs. Gene ontology and kyoto encyclopedia of genes and genomes analyses analyses revealed these targets were mainly associated with metabolic process, HIF-1 signaling pathway, and other biological processes. The competing endogenous RNA network revealed the regulatory relationship between lncRNAs, miRNAs and mRNAs, providing potential targets for the treatment and prevention of diabetic endothelial cell dysfunction. This comprehensive analysis for lncRNAs m6A modification in high glucose and TNF-α-induced human umbilical vein endothelial cells not only demonstrated the understanding of characteristics of endothelial cell dysfunction, but also provided the new targets for the clinical treatment of diabetes. Private information from individuals will not be published. This systematic review also does not involve endangering participant rights. Ethical approval will not be required. The results may be published in a peer-reviewed journal or disseminated at relevant conferences.

## 1. Introduction

Type 2 diabetes mellitus (T2DM), is a chronic endocrine and metabolic disorder syndrome, accounting for more than 90% of diabetic patients.^[1,2]^ It is characterized by hyperglycemia, hyperlipidemia, hypertension and insulin resistance. T2DM, which is common along with complications such as diabetic nephropathy, retinopathy, and vascular disease.^[3,4]^ Accumulating evidence suggested that patients with T2DM have an increased risk of vascular complications. Meanwhile, the development of coronary heart disease, cardiomyopathy, arrhythmias and peripheral artery disease are the primary cause of mortality in T2DM.^[5,6]^ Owing to its complex and diverse pathogenesis, the mechanism of the impairment of cardiovascular disease in diabetes has not been fully established. However, oxidative stress mediated by hyperglycemia and activation of inflammatory damage have been recognized as key underlying events. In addition, many clinical studies have demonstrated that vascular endothelial cell proliferation, basement membrane thickening, and deposition of hyaline-like substances may contribute to the mechanism of diabetic vascular disease.^[7,8]^

Long non-coding RNAs (lncRNAs) are defined as RNA molecules with a transcript length of more than 200 nt. Several studies have shown that lncRNAs participate in many life activities, such as dosage compensation effect, epigenetic regulation, cell cycle and differentiation regulation.^[9,10]^ Abnormal expression of lncRNAs has been found to play an important role in multiple diseases, especially in T2DM and its complications. Although it has gained increasingly deep research, the underlying molecular mechanism of lncRNAs was still mostly uncharacterized in diabetic angiopathies.

N6-methyladenosine (m6A), the most prevalent and abundant internal modification of RNA in eukaryotic cells, which plays essential roles in mRNA metabolism and multiple biological processes.^[11,12]^ The dysregulation of m6A modification is associated with almost every stage of RNA metabolism, ranging from RNA splicing, nuclear export and translation to stability.^[13]^ Current studies have identified that aberrant m6A methylation in diabetic angiopathies, including heart failure, vascular calcification and pulmonary hypertension.^[14–16]^ However, the molecular mechanism of m6A methylation in multifarious diseases is still not fully understood. Therefore, proclaiming the potential of m6A machinery as novel targets for prevention and treatment diabetic angiopathies will attract more attention.

In addition, accumulating research has demonstrated that abundant lncRNAs are also highly modified with m6A to perform their functions. Whereas the pathogenic mechanism of m6A modification lncRNAs in diabetic atherosclerosis have not been comprehensively clarified. In addition, TNF-α as proinflammatory cytokines is an important trigger of endothelial cell dysfunction. Meanwhile, it has been demonstrated they play a crucial role in promoting endothelial cell dysfunction via diverse mechanisms, such as oxidative stress and inflammatory response. Accordingly, high glucose and TNF-α induced endothelial cell dysfunction model in human umbilical vein endothelial cells (HUVECs) was established to reveal the function of m6A modification lncRNAs in atherosclerosis. It also provides a reference for exploring new targets for the diagnosis and treatment of atherosclerosis.

## 2. Materials and Methods

### 2.1. Cell culture and pretreatment

HUVECs were purchased from iCell Biological Technology (Shanghai, China). HUVECs cells were cultured in Dulbecco’s modified Eagle’s medium (DMEM) supplemented with 10% fetal bovine serum (FBS; BI), 100 U/mL penicillin, 100 mg/mL streptomycin at 37°C in 5% CO_2_-humidified incubator. To estimate the effects of high glucose on HUVECs, the cells were pretreated with medium containing 5.5 mM glucose, and then subjected to high glucose and TNF-α treatment. Meanwhile, 25 mM mannitol was used as an osmolarity control condition. Lastly, culture medium concentration was generated by adding 25 mM glucose and 5 ng/mL TNF-α as the final qualification.^[17]^ Afterwards, different treatment cells in two groups were cultured in an incubator for 48 hours for the further experiment.

### 2.2. MeRIP and RNA library preparation and sequencing

Total RNA was extracted using TRIzol reagent (Invitrogen, CA), according to the manufacturer’s protocol. RNA integrity and quality were analyzed via nanodrop and gel electrophoresis. m6A RNA immunoprecipitate was performed by the GenSeqTM m6A-MeRIP Kit (GenSeq Inc., Malaysia), according to the manufacturer’s instructions. Both the input samples without immunoprecipitation and the m6A samples with immunoprecipitation were used for RNA-seq library generation. The library quality was estimated with Bioptic Qsep100 Analyzer (Bioptic lnc., Taiwan, China). MeRIP-Seq service was rendered by Shanghai Biotechnology Corporation (Shanghai, China). Library sequencing was transacted on an illumina NovaSeq 6000 with 150bp paired-end reads.

### 2.3. Data analysis of MeRIP and RNA sequencing

The quality of the original sequencing data was evaluated through FastQC software (v0.11.7). The raw reads were trimmed using Cutadapt (v2.5) and HISAT2 software (v2.1.0) was aligned to the Ensembl database (GRCh38/hg38). Cutadapt (v2.5) was used to trim adapters and filter for sequences, remaining reads were aligned to the human Ensemble database (GRCh38/hg38). The MeRIP enriched peaks were identified using exomePeak (v2.13.2). Differentially m6A-methylated lncRNAs peaks between the control and model group were analyzed using exomepeak software by Poisson–Gamma performed. Identified m6A peaks that *P* < .05 were chosen for the denovo motif analysis using HOMER (v4.10.4).

The gene ontology (GO) and kyoto encyclopedia of genes and genomes (KEGG) enrichment analyses were performed for the differentially methylated related genes. The competing endogenous RNA network was constructed through the Cytoscape software (v3.6.1). The targets of lncRNAs were predicted from the LnaACTdb and LncTarD tools.

## 3. Results

### 3.1. Characteristics of m6A methylation of lncRNAs

**Figure 1.**
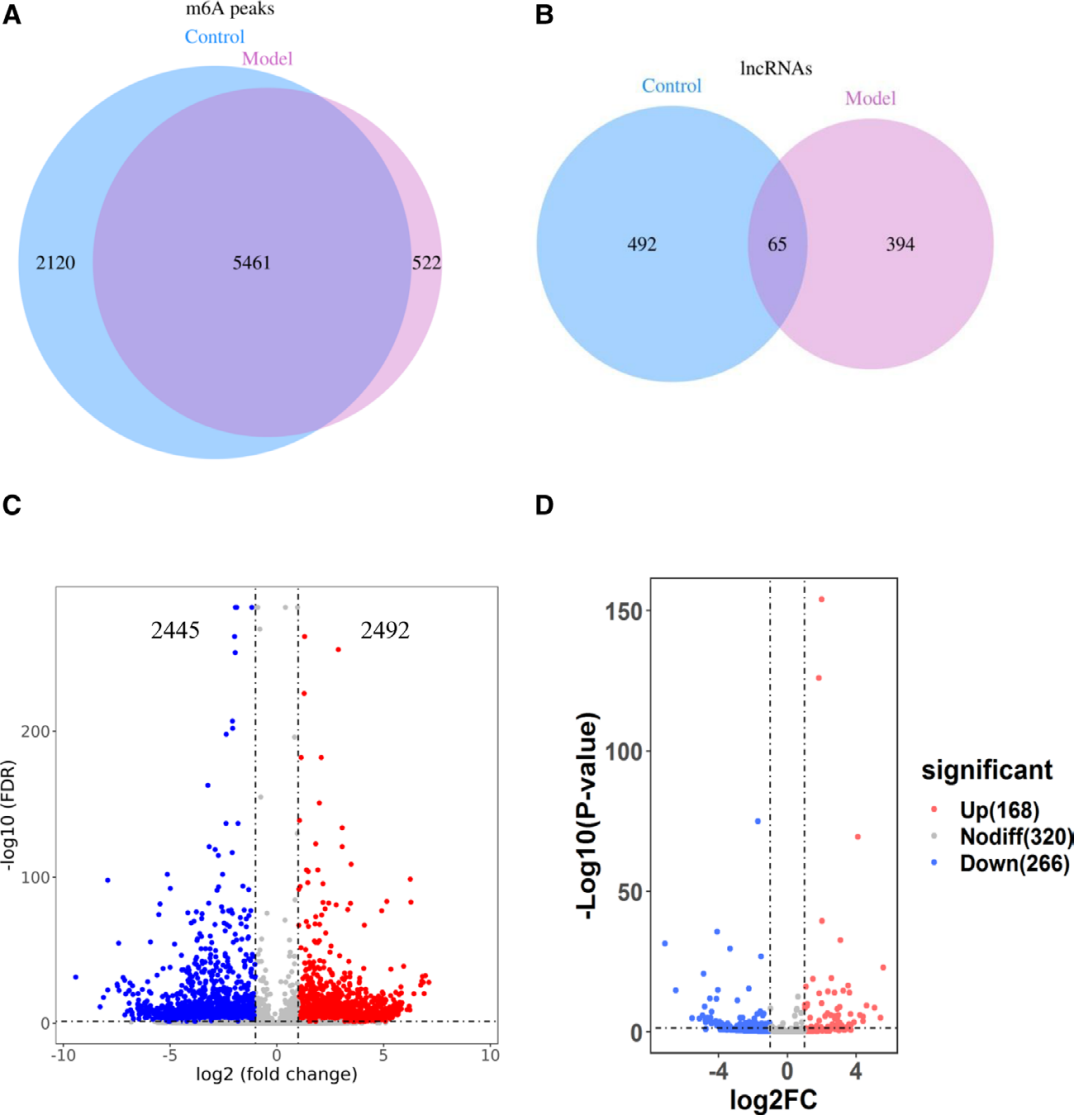
Overview of m6A methylation within lncRNAs in HUVECs. (A) Venn diagram showing the numbers of m6A peaks in the two groups. (B) Venn diagram showing the numbers of lncRNAs in the two groups. (C) Volcano plots displaying the differentially expressed m6A peaks between the two groups. (D) Volcano plots displaying the differentially expressed m6A methylated lncRNAs between the two groups. HUVECs = human umbilical vein endothelial cells, lncRNAs = long non-coding RNAs, m6A = N6-methyladenosine.

A total of 7581 m6A peaks were detected within 557 lncRNAs in control group, while 5983 m6A peaks were recognized within 459 lncRNAs in model group (Fig. 1A and B). In addition, 5461 m6A peaks and 65 lncRNAs were identified in both groups. Incidentally, 4937 m6A peaks were all shared in the two groups, including 2492 up-regulated peaks and 2445 down-regulated peaks (Fig. 1C). Meanwhile, 434 differently m6A methylated lncRNAs were screened, containing 168 up-regulated lncRNAs and 266 down-regulated lncRNAs (Fig. 1D). Overall, these results indicated that the degree of the m6A modification in lncRNAs was higher in the control group than in the model group.

The differently m6A methylated lncRNAs were screened under the conditions of fold change > 2 and *P* < .05. Eventually, 119 significant differentially m6A methylated lncRNAs were selected from 754 lncRNAs, including 60 hypermethylated lncRNAs and 59 hypomethylated lncRNAs. The top 10 hypermethylated or hypomethylated lncRNAs are presented in Table 1.

**Table 1:** Top ten hypermethylated or hypomethylated lncRNAs.

Gene name	Chromosome	*P* value	log_2_FC	Regulation
AC004837.3	7	1.8197E-08	5.11	Hypermethylated
SDCBP2-AS1	20	2.95121E-05	4.53	Hypermethylated
XIST	X	.001548817	4.44	Hypermethylated
HHIP-AS1	4	2.18776E-06	4.43	Hypermethylated
NEAT1	11	.00162181	3.85	Hypermethylated
KLF3-AS1	4	7.94328E-12	3.53	Hypermethylated
AC090772.3	18	.002238721	3.45	Hypermethylated
AC145207.3	17	1.58489E-15	3.38	Hypermethylated
AC079921.1	4	1.86209E-09	3.33	Hypermethylated
NAV2-AS2	11	1.1749E-06	2.81	Hypermethylated
AC020978.6	16	.001174898	−4.61	Hypomethylated
ADAMTS9-AS1	3	1.25893E-11	−4.66	Hypomethylated
LINC02407	12	9.12011E-09	−4.72	Hypomethylated
AC021078.1	5	9.77237E-09	−4.86	Hypomethylated
KF456478.1	19	9.12011E-06	−4.9	Hypomethylated
HAGLR	2	8.70964E-05	−5.11	Hypomethylated
LINC02577	7	.000158489	−5.38	Hypomethylated
AP4B1-AS1	1	6.30957E-13	−5.52	Hypomethylated
LINC00607	2	7.94328E-23	−5.62	Hypomethylated
AC124283.1	17	3.16228E-18	−6.46	Hypomethylated

lncRNAs = long non-coding RNAs.

### 3.2. Distribution of differentially m6A methylated lncRNAs

**Figure 2.**
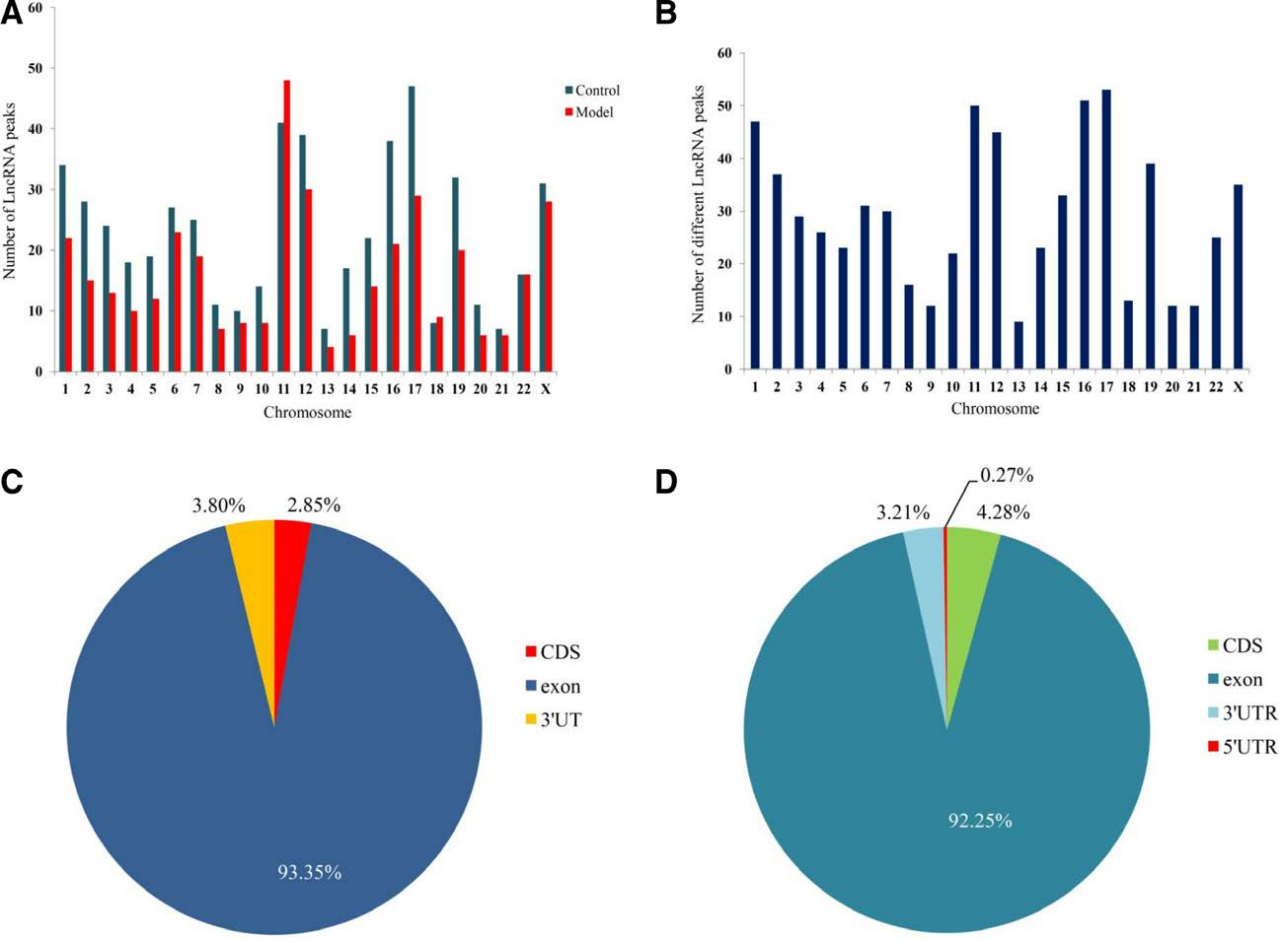
Distribution of differentially m6A methylated lncRNAs. (A) Distribution of m6A modification lncRNAs on chromosomes. (B) Distribution of differentially methylated lncRNAs on chromosomes. (C) and (D) Distribution sites of differentially methylated lncRNAs on chromosomes. CDS = coding sequences, lncRNAs = long non-coding RNAs, m6A = N6-methyladenosine.

To illustrate the distribution of differentially m6A methylated peaks across chromosomes, we analyzed the enrichment level of m6A methylated peaks on each chromosome. The lncRNAs peaks were primarily located on chromosomes 11, chromosomes 12, chromosomes 17, and chromosomes X (Fig. 2A). Meanwhile, hypermethylated lncRNAs peaks were primarily located on chromosomes 11 (15.02%), chromosomes 17 (6.44%), and chromosomes X (8.15%), hypomethylated lncRNAs peaks were principally concentrate on chromosomes 12 (7.16%), chromosomes 16 (9.24%), and chromosomes 17 (8.78%) (Fig. 2B).

Inasmuch to further expose the positional relationship of m6A methylated lncRNAs, we divided into the following categories, containing coding sequences, 3′-untranslated regions (UTRs), 5′-UTRs, and exon. In control group, m6A methylated lncRNAs levels were increased in the exon (93.350% vs 92.25%) and 3′-UTRs (3.80% vs 3.21%), when compared to the model group (Fig. 2C). On the contrary, m6A methylated lncRNAs levels were decreased in coding sequences when compared to the model (2.85 % vs 4.28 %). Moreover, approximately 0.27% of the m6A methylated lncRNAs were only appeared in the 5′-UTRs region of the model group (Fig. 2D).

### 3.3. Abundance of m6A peaks and conserved m6A motifs in lncRNAs

**Figure 3.**
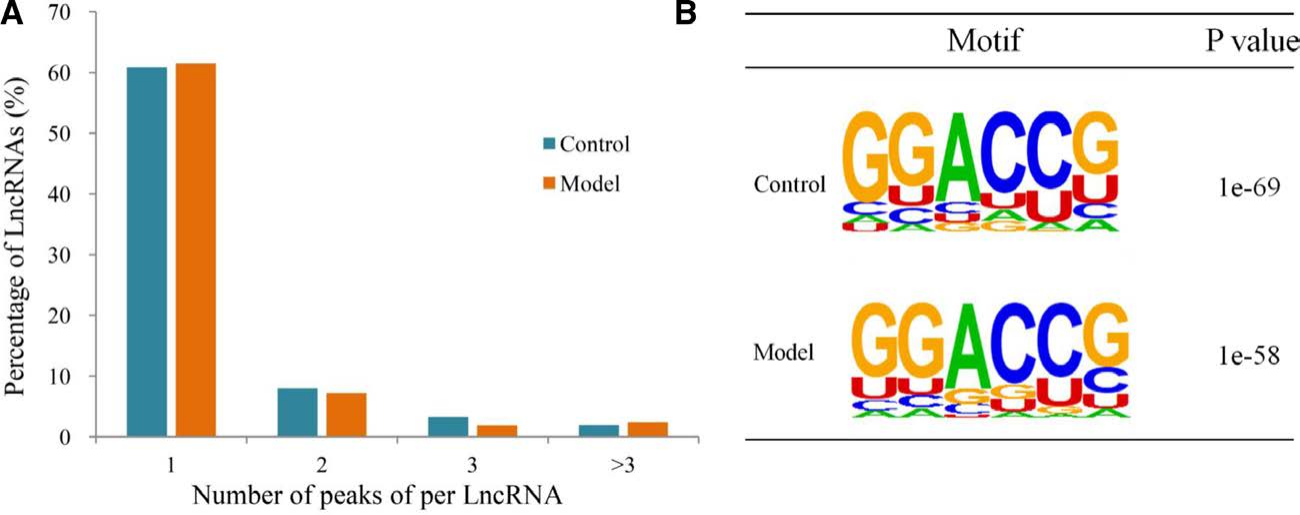
Abundance of m6A peaks and the conserved m6A modified motif in lncRNAs (A) Proportions of lncRNAs harboring different numbers of m6A peaks in the two groups. (B) The sequence motifs of the m6A-containing peak regions in the two groups. lncRNAs = long non-coding RNAs, m6A = N6-methyladenosine.

Regarding the abundance of the m6A peaks in lncRNAs, we found that 77.13% of the lncRNAs in the control group contained m6A peaks, which appeared marginally more than the unimodal value calculated at 75.86% in the model group. The respective percentages comparing different numbers of peaks were also determined with two peaks, three peaks, and more than three peaks being 15.81 versus 16.66, 3.92% versus 5.10 and 3.14% versus 2.38%, respectively, for the control versus model group (Fig. 3A).

To analyze the conserved motif of m6A methylated lncRNAs, we selected the sequences of the peaks with the highest enrichment factor in two groups. The motif sequence was compared with the peaks with the highest enrichment ratio of lncRNA. It was found that GGACCG sequence was one of the conserved motif sequences of lncRNA based on *P* value (Fig. 3B).

### 3.4. Synopsis of differentially expressed lncRNAs in HUVECs

**Figure 4.**
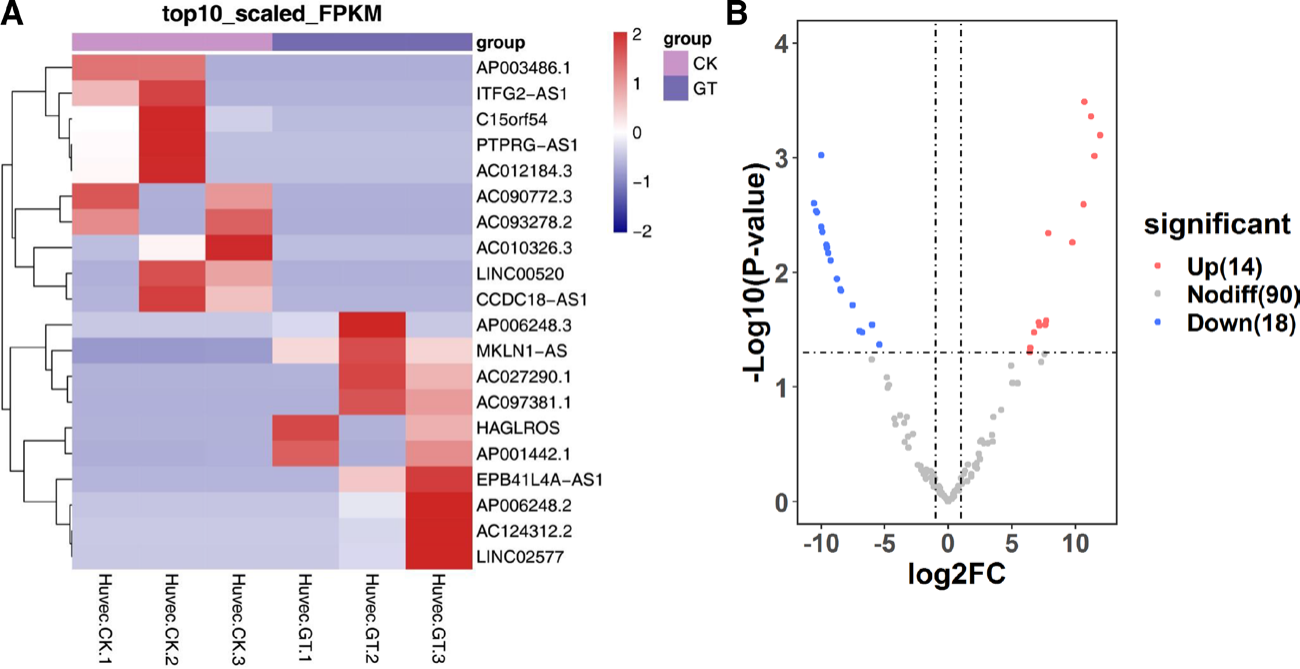
The features of differentially expressed lncRNAs. (A) Hierarchical cluster analysis of differentially expressed lncRNAs in two groups (CK: control; GT: model). (B) General numbers of differentially expressed lncRNAs. lncRNAs = long non-coding RNAs.

As shown in Figure 4A, the results indicated that these lncRNAs have different expression patterns in the two groups. There were 32 significant differentially expressed lncRNAs in HUVECs, which were 14 up-regulated and 266 down regulated (Fig. 4B). The top 10 up-regulated and down-regulated lncRNAs are listed in Table 2.

**Table 2:** Top ten up-regulated and down-regulated expression lncRNAs.

Gene name	log2FoldChange	*P* value	*P*adj	Regulation
EPB41L4A-AS1	10.7124	.0003	.0345	Up
AC027290.1	11.2259	.0004	.0345	Up
AC097381.1	11.9465	.0006	.0369	Up
HAGLROS	11.5021	.0010	.0419	Up
AC124312.2	10.6515	.0026	.0556	Up
MKLN1-AS	7.8622	.0045	.0647	Up
LINC02577	9.7486	.0055	.0674	Up
AP006248.2	7.6929	.0265	.1726	Up
AP001442.1	7.1202	.0272	.1760	Up
AP006248.3	7.6372	.0285	.1816	Up
C15orf54	−10.0005	.0010	.0419	Down
LINC00520	−10.5716	.0025	.0555	Down
ITFG2-AS1	−10.3888	.0029	.0576	Down
AC090772.3	−10.3306	.0030	.0576	Down
AC093278.2	−9.9957	.0040	.0611	Down
AP003486.1	−9.8917	.0044	.0636	Down
CCDC18-AS1	−9.5973	.0058	.0685	Down
PTPRG-AS1	−9.5731	.0061	.0691	Down
AC010326.3	−9.4434	.0068	.0715	Down
AC012184.3	−9.2529	.0078	.0772	Down

lncRNAs = long non-coding RNAs.

### 3.5. Association of m6A methylation and expression of lncRNAs

**Figure 5.**
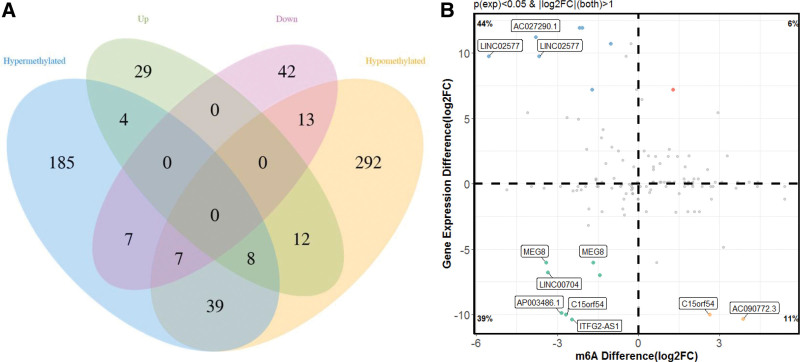
The association between lncRNAs m6A methylation and expression. (A) Venn diagram showing the relationship between m6A modification and expression. (B) Four-quadrant diagram of the relationship between lncRNAs methylation and expression. lncRNAs = long non-coding RNAs, m6A = N6-methyladenosine.

Combining the results of methylation sequencing and RNA sequencing, we found that 185 lncRNAs were hypermethylated, of which was only 4 differentially expressed lncRNAs were up-expressed. A total of 292 lncRNAs were hypomethylated, among them, 13 lncRNAs were down-expressed (Fig. 5A).

To analyze the correlation between lncRNAs methylation level and expression level, a correlation graph was constructed using the fold enrichment of lncRNA m6A methylation and expression value in terms of FPKM. The results demonstrated that there was a statistically significant positive correlation between methylation and expression levels of lncRNAs in the control and model groups (Fig. 5B).

### 3.6. Functional of differentially m6A methylation and expression of lncRNAs

**Figure 6.**
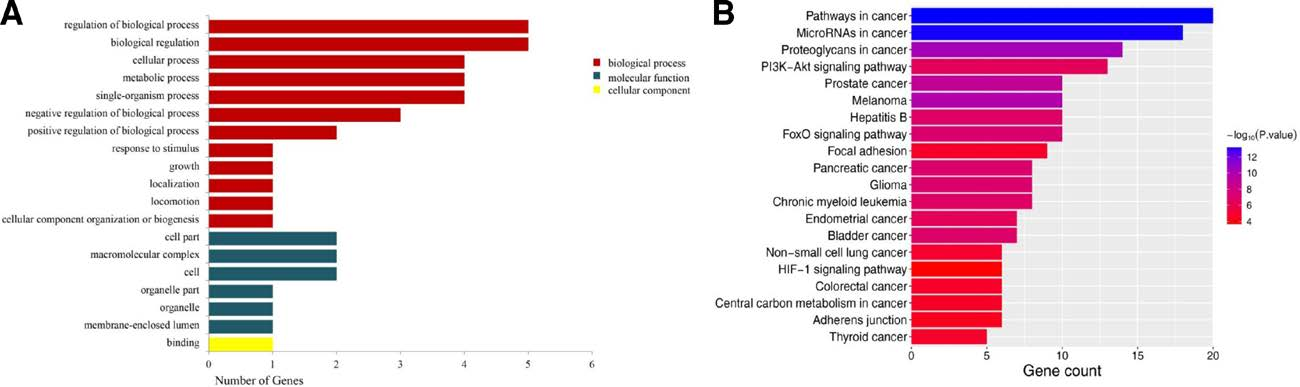
Functional analysis of mRNAs located near differentially methylated lncRNAs. (A) GO enrichment analysis of genes near m6A methylated lncRNAs. (B) KEGG enrichment analysis of genes near m6A methylated lncRNAs. GO = gene ontology, KEGG = kyoto encyclopedia of genes and genomes, lncRNAs = long non-coding RNAs, m6A = N6-methyladenosine.

Owing to manifest the role of differentially methylated and expression of lncRNAs in the occurrence and development of endothelial cell dysfunction, GO and KEGG pathway analyses were performed on the genes located near differentially lncRNAs. GO results revealed that these genes primarily participate in the metabolic process, positive regulation of biological process, biological regulation, and cellular process in the biological process category. In terms of cellular component, genes are associated with cell part, organelle part, and so on. In terms of molecular function, genes are primarily involved in contains binding (Fig. 6A). Meanwhile, we have demonstrated that five lncRNAs MEG3, MALAT1, FTX, XIST, and NEAT1 were mainly concentrated on the GO analysis, which deserves more attention in future research.

KEGG analysis found that the most important signaling pathways associated with genes were significantly enriched in PI3K-Akt signaling pathway, FOXO signaling pathway, pancreatic cancer and HIF-1 signaling pathway (Fig. 6B). These genes, such as PI3K, FOXO, HIF-1α, TGF-1β, MAPK1, EGFR, and BCL2 participated in the function of these pathways and accelerated the degree of endothelial cell dysfunction.

### 3.7. Construction of lncRNA-miRNA-mRNA network

**Figure 7.**
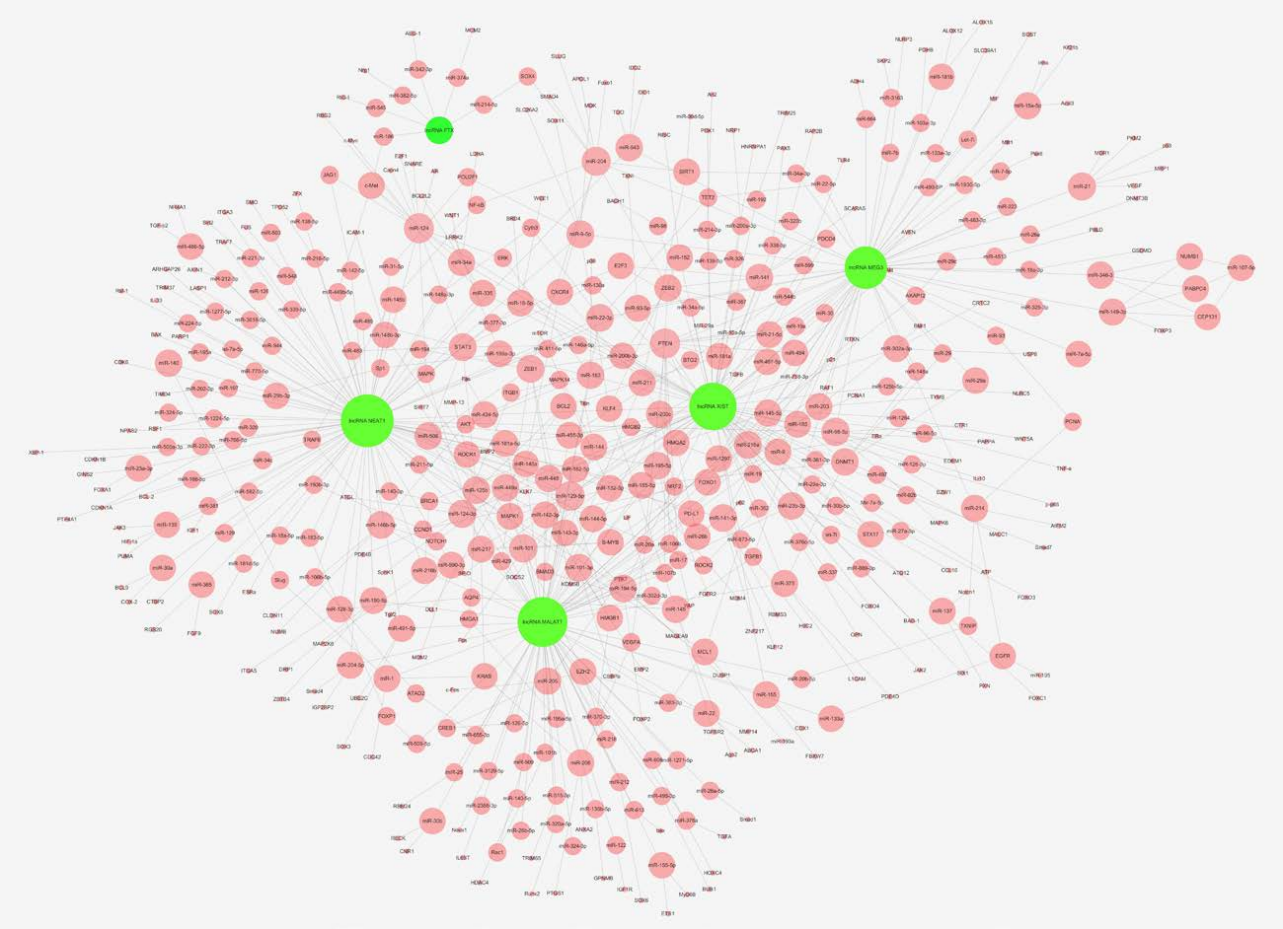
The networks of lncRNA-miRNA-mRNA regulation. Green represent the lncRNAs, genes in red are coding miRNA and mRNAs. lncRNAs = long non-coding RNAs.

Five significant lncRNAs were screened out from 51 differentially methylated and expression lncRNAs, of which were associated with endothelial cell dysfunction. The network of lncRNA-miRNA-mRNA was constructed by Cytoscape software. It consisted of the screened lncRNAs and combined with predicted corresponding miRNAs and mRNAs from the LnaACTdb and LncTarD software. The network contained 5 lncRNAs, 231 miRNAs, and 273 mRNAs (Fig. 7). It is evident from the competing endogenous RNA network that lncRNAs regulate the complex relationship between miRNAs and mRNAs. The network will contribute to understanding of the functions and mechanisms of lncRNAs in endothelial cell dysfunction.

## 4. Discussion

In this research, we have investigated the m6A modification lncRNAs by high glucose and TNF-α influenced in HUVECs. The results performed significantly different between control and model groups with the abundance and distribution of m6A modification. In addition, the ratio of m6A modified lncRNAs in control group was remarkably greater than the model group, which indicated m6A modification down regulation of lncRNA expression in the model group. Meanwhile, previous research demonstrated that METTL14-mediated m6A methylation modification suppressed pyroptosis and diabetic cardiomyopathy by down-regulating TINCR.^[18]^ Interestingly, the m6A methylation of XIST was prominently reduced, but the level of XIST was obviously over expression.^[19]^ Notwithstanding, no studies have authenticated the absolute regulatory relationship between m6A methylation levels and lncRNA expression, which requires further in-depth research.

The targets of m6A methylation lncRNAs were analyzed through GO enrichment, including biological processes, molecular function and cellular components. Biological processes results were mainly involved in metabolic process, positive regulation of biological process, biological regulation, and cellular process. Molecular function enrichment analysis was mostly concentrated on binding. Meanwhile, cellular components consist of macromolecular complex, cell part, and organelle part. These biological processes were mostly related to glucose and lipid metabolism, indicating that m6A modified lncRNAs perhaps involved in regulating insulin secretion and reducing blood glucose. GO analysis results revealed that majority of the m6A modified lncRNAs were hypomethylated sites, of which specific mechanisms still need further investigation.

KEGG pathways demonstrated that most of m6A modified lncRNAs were down-regulated in the model group. As an upstream pathway of FOXO, PI3K/Akt could reduce Akt phosphorylation and activate insulin secretion to reduce the level of blood glucose. Additionally, research suggests that regulating PI3K/Akt pathway alleviates oxidative stress and inhibits apoptosis in diabetic cardiomyopathy, together with attenuated diabetic vasculopathy.^[20,21]^ FOXO signaling pathway could regulate insulin signaling, gluconeogenesis and immune cell migration in diabetes. Previous research has declared that metformin might alleviated HUVECs apoptosis and vascular endothelial injury by regulating FOXO protein.^[22]^ Meanwhile, activated PI3K/Akt might regulate glycolysis through the HIF-1α pathway and reduce vascular damage under hypoxic conditions.^[23]^ Altogether, these m6A modified lncRNAs mediated pathways could harmonize blood glucose, diminish the degree of atherosclerosis and alleviate endothelial cell dysfunction.

In addition, our results manifested that lncRNAs NEAT1, XIST, MALAT1, FTX, and MEG3 were differentially expressed in two groups, which might play a crucial role in m6A modification and endothelial cell dysfunction of HUVECs. NEAT1 has been reported to participate in multiple diabetic metabolic syndrome, which accelerates the occurrence and development of diabetic nephropathy by sponging miR-23c.^[24]^ Others have claimed that lncRNA NEAT1 regulated diabetic retinal epithelial-mesenchymal transition via regulating miR-204/SOX4 axis.^[25]^ Our research demonstrated that the expression level of NEAT1 was significantly higher in the model group, which might be related to m6A modification. LncRNA XIST was downregulated in high glucose treated podocytes, accompanied with increased apoptosis of podocytes. Furthermore, lncRNA XIST protects podocyte from high glucose-induced cell injury by sponging miR-30 and regulating AVEN expression in diabetic nephropathy.^[26]^ Additionally, m6A modification of lncRNAs levels has been observed in various prime aggravators of diabetic nephropathy pathogenesis.^[27]^ The targets of XIST were involved in BCL2, STAT3, MAPK1, and VEGF, which mainly concentrate on the immune regulation, oxidative stress, inflammatory response.

What’s more, current studies suggest that the lncRNAs NEAT1, XIST, and MALAT1 participate in the pathogenesis of T2DM and highlight their potential as diagnostic biomarkers, especially MALAT1, which is highly expressed in the serum of patients with coronary atherosclerosis heart disease, and it has high value in the diagnosis and prediction of in-stent restenosis.^[28]^ Inhibition of MALAT1 has the potential to protect the retina from oxidative damage and to prevent diabetic retinopathy.^[29]^ FTX has been reported to be associated with several tumor progressions, such as hepatocellular carcinoma,^[30]^ renal cell carcinoma,^[31]^ and colorectal cancer.^[32]^ Nevertheless, we have revealed that it was associated with endothelial cell dysfunction, and further experiments are required and validate their effects and mechanisms. LncRNA MEG3 is related with multiple biological processes, containing proliferation, apoptosis and inflammation response. Previous research has illustrated MEG3 could alleviate high glucose inducing apoptosis and inflammation via inhibiting NF-κB pathway by targeting miR-34a/SIRT1 axis.^[33]^ In addition, ROCK2, TNF, NOTCH1, and HIF-1α have also played crucial regulatory roles during atherogenesis. Accordingly, controlling blood glucose and reducing the stimulation of inflammatory factors have obvious promoting effects on reducing vascular damage and improving atherosclerosis.

However, our study had a few limitations regarding the analysis of m6A-sequencing and RNA-sequencing. All the results were only based on association studies and bioinformatic analyses, which need further experiments to verify the results. In addition, the functions of m6A modification lncRNAs and their targets related to endothelial dysfunction require further investigation.

## 5. Conclusion

In summary, we firstly established a comprehensive analysis to investigate the m6A modification of lncRNAs in HUVECs to predict the mechanism of high glucose and TNF-α induced endothelial cell dysfunction. Despite the above limitations, our research still provides a new theoretical basis for the study of diabetic endothelial cell dysfunction, as well as new targets and reference for clinical treatment of atherosclerosis.

## Author contributions

**Conceptualization:** Jiarong Gao.

Data curation: Wei Zhang.

Funding acquisition: Jiarong Gao.

Investigation: Liangbing Wei.

Methodology: Liangbing Wei, Wei Zhang.

Project administration: Jiarong Gao.

Resources: Li Shan.

Software: Mingfei Guo, Yaji Dai.

Validation: Mingfei Guo, Yaji Dai.

Writing – original draft: Li Shan, Mingfei Guo.

Writing – review & editing: Yaji Dai.

## References

[1] Ferguson D Finck BN. Emerging therapeutic approaches for the treatment of NAFLD and type 2 diabetes mellitus. Nat Rev Endocrinol. 2021;17:484–95. 34131333 10.1038/s41574-021-00507-z PMC8570106

[2] Nauck MA Wefers J Meier JJ. Treatment of type 2 diabetes: challenges, hopes, and anticipated successes. Lancet Diabetes Endocrinol. 2021;9:525–44. 34181914 10.1016/S2213-8587(21)00113-3

[3] Demir S Nawroth PP Herzig S. Emerging targets in type 2 diabetes and diabetic complications. Adv Sci (Weinh). 2021;8:e2100275. 34319011 10.1002/advs.202100275 PMC8456215

[4] Seewoodhary M. An overview of diabetic retinopathy and other ocular complications of diabetes mellitus. Nurs Stand. 2021;36:71–6. 10.7748/ns.2021.e11696 34032037

[5] Viigimaa M Sachinidis A Toumpourleka M. Macrovascular complications of type 2 diabetes mellitus. Curr Vasc Pharmacol. 2020;18:110–6. 30961498 10.2174/1570161117666190405165151

[6] Yun JS Ko SH. Current trends in epidemiology of cardiovascular disease and cardiovascular risk management in type 2 diabetes. Metabolism. 2021;123:154838. 34333002 10.1016/j.metabol.2021.154838

[7] Su L Kong X Loo S. Thymosin beta-4 improves endothelial function and reparative potency of diabetic endothelial cells differentiated from patient induced pluripotent stem cells. Stem Cell Res Ther. 2022;13:13. 35012642 10.1186/s13287-021-02687-x PMC8751378

[8] Zhu D Zhang X Wang F. Irisin rescues diabetic cardiac microvascular injury via ERK1/2/Nrf2/HO-1 mediated inhibition of oxidative stress. Diabetes Res Clin Pract. 2022;183:109170. 34863716 10.1016/j.diabres.2021.109170

[9] Yan S Sun M Gao L. Identification of key LncRNAs and pathways in prediabetes and type 2 diabetes mellitus for hypertriglyceridemia patients based on weighted gene co-expression network analysis. Front Endocrinol (Lausanne). 2022;12:800123. 35140684 10.3389/fendo.2021.800123 PMC8818867

[10] Lan X Han J Wang B. Integrated analysis of transcriptome profiling of lncRNAs and mRNAs in livers of type 2 diabetes mellitus. Physiol Genomics. 2022;54:86–97. 35073196 10.1152/physiolgenomics.00105.2021

[11] Chen YS Ouyang XP Yu XH. N6-adenosine methylation (m^6^A) RNA modification: an emerging role in cardiovascular diseases. J Cardiovasc Transl Res. 2021;14:857–72. 33630241 10.1007/s12265-021-10108-w

[12] Zhong H Tang HF Kai Y. N6-methyladenine RNA modification (m^6^A): an emerging regulator of metabolic diseases. Curr Drug Targets. 2020;21:1056–67. 32066359 10.2174/1389450121666200210125247

[13] Huang J Yin P. Structural insights into N^6^-methyladenosine (m^6^A) modification in the transcriptome. Genomics Proteomics Bioinformatics. 2018;16:85–98. 29709557 10.1016/j.gpb.2018.03.001 PMC6112310

[14] Berulava T Buchholz E Elerdashvili V. Changes in m6A RNA methylation contribute to heart failure progression by modulating translation. Eur J Heart Fail. 2020;22:54–66. 31849158 10.1002/ejhf.1672

[15] Qin Y Qiao Y Li L. The m^6^A methyltransferase METTL3 promotes hypoxic pulmonary arterial hypertension. Life Sci. 2021;274:119366. 33741419 10.1016/j.lfs.2021.119366

[16] Xu S Xu X Zhang Z. The role of RNA m^6^A methylation in the regulation of postnatal hypoxia-induced pulmonary hypertension. Respir Res. 2021;22:121. 33902609 10.1186/s12931-021-01728-6 PMC8074209

[17] Calandrelli R Xu L Luo Y. Stress-induced RNA-chromatin interactions promote endothelial dysfunction. Nat Commun. 2020;11:5211. 33060583 10.1038/s41467-020-18957-w PMC7566596

[18] Meng L Lin H Huang X. METTL14 suppresses pyroptosis and diabetic cardiomyopathy by downregulating TINCR lncRNA. Cell Death Dis. 2022;13:38. 35013106 10.1038/s41419-021-04484-z PMC8748685

[19] Yang X Zhang S He C. METTL14 suppresses proliferation and metastasis of colorectal cancer by down-regulating oncogenic long non-coding RNA XIST. Mol Cancer. 2020;19:46. 32111213 10.1186/s12943-020-1146-4 PMC7047419

[20] Ren BC Zhang YF Liu SS. Curcumin alleviates oxidative stress and inhibits apoptosis in diabetic cardiomyopathy via Sirt1-Foxo1 and PI3K-Akt signalling pathways. J Cell Mol Med. 2020;24:12355–67. 32961025 10.1111/jcmm.15725 PMC7687015

[21] Liu Y Wei J Ma KT. Carvacrol protects against diabetes-induced hypercontractility in the aorta through activation of the PI3K/Akt pathway. Biomed Pharmacother. 2020;125:109825. 32036208 10.1016/j.biopha.2020.109825

[22] Chen L Yin Y Liu G. Metformin alleviates bevacizumab-induced vascular endothelial injury by up-regulating GDF15 and activating the PI3K/AKT/FOXO/PPARγ signaling pathway. Ann Transl Med. 2021;9:1547. 34790753 10.21037/atm-21-4764 PMC8576656

[23] Pan T Sun S Chen Y. Immune effects of PI3K/Akt/HIF-1α-regulated glycolysis in polymorphonuclear neutrophils during sepsis. Crit Care. 2022;26:29. 35090526 10.1186/s13054-022-03893-6 PMC8796568

[24] Li N Jia T Li YR. LncRNA NEAT1 accelerates the occurrence and development of diabetic nephropathy by sponging miR-23c. Eur Rev Med Pharmacol Sci. 2020;24:1325–37. 32096162 10.26355/eurrev_202002_20190

[25] Yang Y Zhou J Li WH. LncRNA NEAT1 regulated diabetic retinal epithelial-mesenchymal transition through regulating miR-204/SOX4 axis. PeerJ. 2021;9:e11817. 34386303 10.7717/peerj.11817 PMC8312494

[26] Long B Wan Y Zhang S. LncRNA XIST protects podocyte from high glucose-induced cell injury in diabetic nephropathy by sponging miR-30 and regulating AVEN expression. Arch Physiol Biochem. 2020;17:1–8. 10.1080/13813455.2020.1854307 33332155

[27] Kumari N Karmakar A Ahamad Khan MM. The potential role of m6A RNA methylation in diabetic retinopathy. Exp Eye Res. 2021;208:108616. 33979630 10.1016/j.exer.2021.108616

[28] Qiu S Sun J. lncRNA-MALAT1 expression in patients with coronary atherosclerosis and its predictive value for in-stent restenosis. Exp Ther Med. 2020;20:129. 10.3892/etm.2020.9258 PMC7557524 33082861

[29] Radhakrishnan R Kowluru RA. Long noncoding RNA MALAT1 and regulation of the antioxidant defense system in diabetic retinopathy. Diabetes. 2021;70:227–39. 33051272 10.2337/db20-0375 PMC7881848

[30] Wu H Zhong Z Wang A. LncRNA FTX represses the progression of non-alcoholic fatty liver disease to hepatocellular carcinoma via regulating the M1/M2 polarization of Kupffer cells. Cancer Cell Int. 2020;20:266. 32595415 10.1186/s12935-020-01354-0 PMC7315496

[31] Lin Y Shen Y Chen J Hu C. The function of LncRNA FTX in several common cancers. Curr Pharm Des. 2021;27:2381–6. 33121404 10.2174/1381612826666201029164036

[32] Chen GQ Liao ZM Liu J. LncRNA FTX promotes colorectal cancer cells migration and invasion by miRNA-590-5p/RBPJ axis. Biochem Genet. 2021;59:560–73. 33389283 10.1007/s10528-020-10017-8

[33] Tong P Peng QH Gu LM. LncRNA-MEG3 alleviates high glucose induced inflammation and apoptosis of retina epithelial cells via regulating miR-34a/SIRT1 axis. Exp Mol Pathol. 2019;107:102–9. 30529346 10.1016/j.yexmp.2018.12.003

